# Impact of Static Myoblast Loading on Protein Secretion Linked to Tenocyte Migration

**DOI:** 10.1021/acs.jproteome.5c00068

**Published:** 2025-04-09

**Authors:** Junhong Li, Xin Zhou, Jialin Chen, Shaochun Zhu, Andre Mateus, Pernilla Eliasson, Paul J. Kingham, Ludvig J. Backman

**Affiliations:** † Department of Medical and Translational Biology, UmeÅ University, 90187 UmeÅ, Sweden; ‡ Department of Community Medicine and Rehabilitation, Physiotherapy, UmeÅ University, 90187 UmeÅ, Sweden; § School of Medicine, Southeast University, 210009 Nanjing, China; ∥ Department of Ophthalmology, Zhongda Hospital, Southeast University, 210009 Nanjing, China; ⊥ Department of Chemistry, Umeå University, 90187 Umeå, Sweden; # The Laboratory for Molecular Infection Medicine Sweden, Umeå University, 90187 Umeå, Sweden; ∇ Department of Orthopedics, Sahlgrenska University Hospital, 43180 Gothenburg, Sweden

**Keywords:** static loading, myokines, tenocyte, wound healing, migration

## Abstract

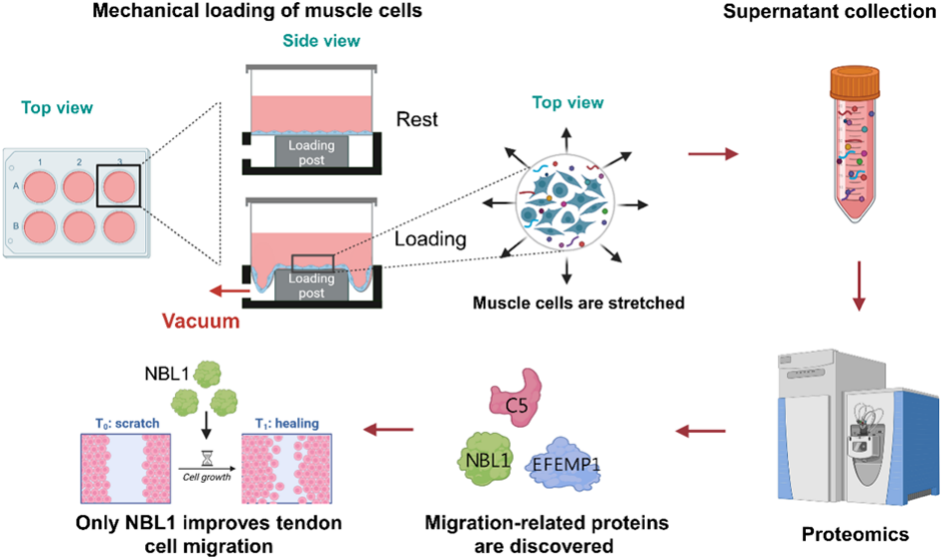

Exercise has been shown to promote wound healing, including tendon repair. Myokines released from the exercised muscles are believed to play a significant role in this process. In our previous study, we used an in vitro coculture and loading model to demonstrate that 2% static loading of myoblasts increased the migration and proliferation of cocultured tenocytes—two crucial aspects of wound healing. IGF-1, released from myoblasts in response to 2% static loading, was identified as a contributor to the increased proliferation. However, the factors responsible for the enhanced migration remained unknown. In the current study, we subjected myoblasts in single culture conditions to 2, 5, and 10% static loading and performed proteomic analysis of the cell supernatants. Gene Ontology (GO) analysis revealed that 2% static loading induced the secretion of NBL1, C5, and EFEMP1, which is associated with cell migration and motility. Further investigation by adding exogenous recombinant proteins to human tenocytes showed that NBL1 increased tenocyte migration but not proliferation. This effect was not observed with treatments using C5 and EFEMP1.

## Introduction

Exercise-induced secretory factors are believed to play a crucial role in mediating the health benefits of physical activity. These secreted factors (termed myokines) are believed to originate, at least in part, from muscle tissue, entering the circulatory system and exerting autocrine and endocrine functions. For example, postexercise increases in serum interleukin-6 (IL-6) concentrations are accompanied by elevated IL-6 mRNA expression in muscle tissue.^1^ Steensberg et al. confirmed that contracting muscles produce IL-6, accounting for the exercise-induced plasma IL-6 increase, by comparing arterial-femoral venous concentrations in exercising versus resting legs.^2^ Similarly, increased serum levels of insulin-like growth factor-1 (IGF-1)^3,4^ and interleukin-15 (IL-15)^5^ have also been shown to originate from exercised muscle tissue.

Skeletal muscle fibers sense and transmit mechanical stimuli through their sarcolemma ion channels, dystrophin-glycoprotein complex (DGC), integrins/vinculin/talin complex, sarcomere giant protein titin, cellular cytoskeleton, and nuclear LINC complex. These mechanical stimuli are ultimately conveyed through signaling pathways such as ERK, mTORC1, and p70S6K, influencing gene expression profiles and thereby regulating cellular processes, including protein secretion.^6−8^ Different types, intensities, and durations of exercise can release distinct profiles of myokines into the circulation,^9−11^ exerting varied functions. For example, eccentric exercise increased the serum level of IL-6 after exercise,^12,13^ whereas handball exercise significantly decreased IL-6 levels.^14^ A meta-analysis revealed that exercise interventions lasting more than 12 weeks significantly reduced IL-6 levels.^15^ Similarly, serum IGF-1 levels have been reported to significantly increase in response to strength training, endurance training, badminton, and cycling.^3,4^ However, some studies have reported a decline or no significant effect of resistance training on serum IGF-1 levels.^16^ In addition, by analyzing the human plasma proteome, Guseh et al. found that moderate-intensity exercise influenced plasma concentrations of 184 distinct proteins, while high-intensity exercise affected 598 proteins.^17^ Similarly, in rodents, multiplex assays for mouse serum cytokines showed different sets of cytokines activated in response to a single bout of sprint, endurance running, and one-week voluntary wheel running.^18^ Therefore, these findings highlight the necessity of investigating specific exercise parameters to optimize the generation of beneficial myokine profiles.

In vitro loading systems allow researchers to apply a mechanical load to cultured myoblasts, thereby mimicking the conditions of exercised muscle in vivo and simulating the effects of exercise. Myokines released by mechanically loaded myoblasts play a crucial role in mediating the beneficial effects through both autocrine and paracrine functions. For instance, fibroblast growth factor-2 (FGF2) secretion by mechanically loaded myoblasts is a critical autocrine mechanism for converting mechanical load stimuli into skeletal muscle growth.^19^ Mechanical loading of cultured rat muscle satellite cells accelerates their entry into the cell cycle by releasing hepatocyte growth factor (HGF) into the culture medium.^20^ Tumor necrosis factor-α (TNF-α) derived from the mechanical loading of myoblast mediates mechanotransduction-induced myogenesis.^21^ Furthermore, mechanical loading promotes L6 myoblast proliferation by stimulation of the IGF-1 receptor.^22^ The autocrine function can also be exerted through extracellular vesicles. A study demonstrated that both high and low intensities of mechanical loading on myoblast increased exosome concentrations, leading to greater proliferation and differentiation of myoblasts.^23^

In vitro studies investigating the paracrine and endocrine roles of myokines have primarily focused on the conditioned medium from unloaded muscle cells. There are limited studies investigating how myokines released during mechanical loading influence cells from other tissues. One study demonstrated that myokines derived from mechanically loaded myoblasts promote neutrophil chemotaxis.^24^ In our previous studies, applying a coculture system of tenocytes with mechanically loaded myoblasts, we found that static loading of myoblast-released myokines increased tenocyte proliferation, migration, collagen I/III ratio, and the expression of healing-related markers when compared with dynamically loaded myoblasts.^25,26^ By using RNA-sequencing, we identified IGF-1 as the specific myokine secreted during static loading of myoblast, which exerted a proliferative effect on tenocytes.^26^ However, gene ontology analysis of the RNA-sequencing data did not reveal which factor(s) is responsible for the increased migration of tenocytes. Therefore, in this study, we applied different intensities of static loading to single-cultured myoblasts, collected the supernatants, and performed proteomic analysis to profile the myokines released under different intensities. Gene ontology analysis revealed that under 2% static loading, myoblasts released secretory factors, including neuroblastoma suppressor of tumorigenicity 1 (NBL1), the complement C5 (C5), and EGF-containing fibulin-like extracellular matrix protein 1 (EFEMP1), and proteins involved in regulating cell migration and motility. To further understand the role of these proteins, we investigated their effects on tenocyte migration and found that NBL1 significantly promotes tenocyte migration, whereas C5 and EFEMP1 showed no effect.

## Materials and Methods

### Myoblast Cell Preparation

The L6 rat myoblast cell line was purchased from the American Type Culture Collection (ATCC). Myoblasts were seeded onto FlexCell plates (FlexCell International Corporation, BF-3001C) at a density of 3 × 10^5^ cells per well in 2 mL of Dulbecco’s modified Eagle medium (DMEM)+GlutaMAX (Gibco, 61965-026), supplemented with 10% fetal bovine serum (FBS) (Sigma-Aldrich, 0001648078). Following 48 h of cultivation in a 5% CO_2_ cell culture incubator, the culture medium was aspirated, and the myoblasts were subjected to two washes with Hanks’ Balanced Salt Solution (HBSS). Subsequently, 2.5 mL of skeletal muscle cell growth basal medium-2 (CC-3246, Lonza), supplemented with Singlequots and growth factors (excluding FBS) (CC-3244, Lonza), was added to the myoblast cultures in the FlexCell plates.

### Tenocyte Cell Preparation

We established human primary tenocyte (tendon cells) culture derived from residual tissues of m. semitendinosus obtained during anterior cruciate ligament reconstruction surgery. The Swedish Ethical Review gave ethical permission (dnr 2021-05627-01). All tissue donors signed a written consent form. The tendons obtained from the remnants of surgeries were washed with HBSS, and any attached muscle tissue was carefully removed. The tendons were then cut into small pieces and digested overnight with 2 mg/mL collagenase (Sigma-Aldrich, C0130) dissolved in DMEM + GlutaMAX (Gibco, 61965-026) supplemented with 10% FBS (Sigma-Aldrich, 0001648078) in a cell culture incubator. After digestion, the resulting cell suspension was centrifuged at 500*g* for 5 min. The cell pellets were resuspended and cultured in DMEM + GlutaMAX (Gibco, 61965-026) supplemented with 10% FBS (Sigma-Aldrich, 0001648078) until they reached approximately 80% confluency before passage. Cells at passages 4–5 were used for the experiments. Primary tenocytes were generated following the same established protocol used in our previous studies, where their phenotypes were characterized.^25^ In the current study, tenocyte phenotypes were further assessed using gene markers including Scleraxis (SCX), Tenomodulin (TNMD), Collagen Type I α 1 Chain (COL1A1), Collagen Type III α 1 Chain (COL3A1), and Fibronectin 1 (FN1).

### Mechanical Loading of Myoblast

The FlexCell tension system (FlexCell International Corporation, FX5000) was used to apply mechanical loading to the L6 myoblast. The myoblasts were seeded onto FlexCell plates equipped with elastic culturing membranes. These plates were placed on 25 mm diameter round loading posts and subjected to vacuum suction, enabling an equal biaxial loading to the adherent cells. The membranes of the FlexCell plates were statically elongated by 2, 5, and 10% over a 24 h period, incorporating rest intervals to avoid stimulus adaptation and preserve the mechanical sensitivity of the cells. The selection of the loading intensities was based on existing studies, which demonstrated that loading up to 20% did not cause cell death.^27,28^ No significant morphological changes were observed in this study following the loading. Additionally, the lactate dehydrogenase (LDH) assay conducted in our previous work confirmed that all three intensities of mechanical loading did not induce cell death.^29^ The loading regime consisted of two cycles of one h of loading followed by two h of rest, followed by an additional 1 h loading and 6 h of rest. This process was repeated cyclically for a total duration of 24 h, as described in our previous studies.^25,26^

### Supernatant Collection and Concentration

Following mechanical loading, a total of 13.5 mL of myoblast supernatant was pooled together from six individual wells from one plate. Three plates for each group (*N* = 3) were utilized. The control was cultured under similar circumstances but kept unloaded. The collected supernatant was subjected to centrifugation at 1000*g* for 5 min at 4 °C to eliminate cell debris. To enhance the concentration of supernatant proteins, the supernatants were transferred to protein concentrator tubes (88525S, Thermo Scientific) and subsequently centrifuged at 3100*g* for 4 h at 4 °C. After the concentration step, the samples were then subjected to a triple wash with 2 mL of PBS, accomplished by centrifugation at 3100*g* at 4 °C for durations of 1, 1.5, and 3 h, respectively. Protein concentrations were assessed utilizing the BCA protein assay kit (Thermo Scientific, 23225) according to the manufacturer’s protocol.

### Proteomics

#### Sample Preparation

Samples were subjected to peptide digestion using a modified SP3 protocol.^30^ In brief, the supernatant proteins were reconstituted in a lysis buffer containing 2% SDS and 20 mM TCEP. The mixtures were then heated to 95 °C and maintained at that temperature for 10 min. A 1:1 (v/v) combination of SpeedBeads magnetic carboxylate-modified particles (Sigma-Aldrich, beads A hydrophobic, catalog no. GE45152105050250; beads B hydrophobic, catalog no. GE65152105050250,) was prepared and subjected to four washes using LC-MS water. Subsequently, the beads were mixed with each sample in a binding buffer composed of 50% ethanol and 2.5% formic acid, followed by incubation with gentle shaking at 500 rpm for 15 min at room temperature. The resultant mixtures were then transferred into a filter plate (0.22 μm, Sigma-Aldrich, part.no: MSGVN2210) to remove the unbound fraction via centrifugation at 1000*g*. The Beads were retained on the filter and subsequently washed four times with 70% ethanol. For digestion, trypsin was combined with a digestion solution containing 100 mM HEPES at pH 7.5, 5 mM chloroacetamide, and 1.2 mM TCEP. This trypsin-containing solution was added to each sample on the plate at a ratio of 1 μg of trypsin per 25 μg of protein. The samples were digested overnight at room temperature with gentle shaking at 500 rpm. The resulting flowthrough, containing peptides, was collected through centrifugation at 1000*g*. Subsequently, 10 μL of 2% DMSO was introduced to beads to elute bound peptides, which were then pooled with the previous flowthrough. To desalt the peptides, an Oasis HLB plate (Waters, catalog no. 186001828BA) was employed following the manufacturer’s protocol. The desalted peptides were subsequently dried by using a speed vacuum concentrator.

#### Liquid Chromatography-Tandem Mass Spectrometry (LC–MS/MS) Analysis

Dried peptides were reconstituted with 0.1% formic acid in water. A quantity of 1 μg of peptides from each sample was introduced to a mass spectrometer (MS) using a Vanquish Neo system (Thermo Scientific). The trapping column employed was the PEPMAP NEO C18 (5 μm particle size, 300 μm x 5 mm, Thermo Scientific). The analytical column utilized was the nano EaseTM M/Z HSS C18 T3(100Å, 1.8 μm particle size, 75 μm*250 mm, Waters). A total separation and elution time of 120 min was executed, involving a gradient of mobile phase A (water and 0.1% formic acid) transitioning to 8% of mobile phase B (80% acetonitrile and 0.1% formic acid) over a span of 4 min. This was further followed by a gradual increase to 27% of mobile B over 87 min. Subsequently, there was a rapid rise to 80% of mobile phase B within 6 s, maintained for minutes, and then a final decrease to 2% of mobile phase B within 30 s to conclude the column equilibration.

Data acquisition on the Exploris 480 system (Thermo Scientific) was achieved by using a data-dependent method. Survey scans spanning the mass range of 375–1500 were acquired at a resolution of 120,000, with a radiofrequency (RF) lens setting of 40% and normalized automatic gain control (AGC) set to 300%. A maximum cycling time of 2 s was applied to regulate the number of precursor ions selected for tandem-MS/MS (MS2) analysis. Charge states encompassing 2 to 6 charges were considered. Dynamic exclusion was configured to exclude the previously selected precursor ions for a duration of 35 s. MS2 scans were performed at a resolution of 15,000 (at *m*/*z* 200), with the AGC target value set to auto. An isolation window of 1.4 *m*/*z* was utilized. HCD fragmentation was induced with a normalized collision energy (NCE) of 30, and isotopes were excluded from the MS2 analysis.

#### Data Analysis

The raw data underwent a search against *Rattus norvegicus* UniProt FASTA (proteome identifier [ID] UP000002494). For label-free quantification, the LFQ-MBR workflow MSFragger was employed.^31^ Subsequently, proteins that were identified as contaminants or decoyed were eliminated from consideration. Only those proteins quantified in more than one replicate within each experimental group were selected for further analysis. The statistical analysis was conducted using R (version 4.2.2). To mitigate technical variation, the data was normalized using the vsn package. The evaluation of protein differential expression was performed using the limma package. Differences in protein abundances were statistically assessed through the application of the Student’s *t* test, moderated by Benjamini–Hochberg’s method, to ensure robust results. Two criteria were employed to identify proteins with significant secretion: Proteins showing a fold change greater than 2 and a *p*-value less than 0.05 were classified as increased in abundance, while proteins with a fold change less than 0.5 and a *p*-value less than 0.05 were categorized as decreased in abundance.

### Secretary Protein Prediction

The protein sequences for each differentially secreted protein were provided in Fasta format for analysis. SignalP-6.0 (https://dtu.biolib.com/SignalP-6) was utilized to predict the presence of signal peptides in these proteins. Additionally, SecretomeP-2.0 (https://services.healthtech.dtu.dk/services/SecretomeP-2.0) was employed to predict if these proteins exhibit nonclassical secretion. In SignalP-6.0, the prediction results were represented by a numerical value within the Sec/SPI term ranging from 0 to 1. This number indicated the proportion of Sec/SPI signal peptides (typified by their transport via the Sec translocon and cleavage by Signal Peptidase I (Lep)) compared to all types of signal peptides. A value of 0 indicates the absence of a signal peptide within the protein sequence. In SecretomeP- 2.0, mammalian protein predictions were conveyed using the term NN-score, with a suggested threshold of 0.6 for mammalian sequences. Proteins achieving an NN-score exceeding 0.6 were deemed nonclassically secreted proteins, as shown in our results.

To identify whether predicted nonsecretory proteins could be secreted via extracellular vesicles, the gene symbols of these proteins were cross-referenced with the Vesiclepedia (http://www.microvesicles.org/) database. The terms *Rattus norvegicus*, *Mus musculus*, and *Homo sapiens* under the species category elucidated which species could secrete the protein via extracellular vesicles.

### Pathway and Process Enrichment Analysis

The gene lists of up- and downregulated proteins within each group were separately input into the Database for annotation, visualization, and integrated discovery (DAVID, https://david.ncifcrf.gov/) for the analysis of biological process enrichment. *Rattus norvegicus* was selected as the chosen species. Based on gene ratio considerations, we selected the top ten pathways from Biology process group 5 in Gene Ontology (GO) and Kyoto Encyclopedia of Genes and Genomes (KEGG) pathways to be shown in our figure.

### Tenocyte Cell Migration

Human tenocytes from three different donors were seeded in triplicate for each donor in 12-well plates at a density of 1 × 10^5^ cells per well. After 24 h of incubation, three scratches were made per well using a 200 μL pipet tip. The cells were then washed twice with HBSS and subsequently cultured in DMEM+GlutaMAX (Gibco, 61965-026) medium supplemented with 1% FBS (Sigma-Aldrich, 0001648078), containing varying concentrations of NBL1(R&D, 955-DA), C5(R&D, 2037C5), and EFEMP1(R&D, 8416-FB). The 12-well plates were then placed in an Incucyte S3 live-cell analysis system (Sartorius) to capture images at 4× magnification at 0 and 24 h. Four images were analyzed to measure the mean scratch area per well. The scratch areas were quantified using the wound healing size tool plugin in the ImageJ. The migration rate for each image was calculated by subtracting the wound area size at 24 h from the size at 0 h and then dividing the difference by the area size at 0 h.

### Tenocyte Proliferation

Human tenocytes from three donors were seeded in four replicates for each donor into 96-well plates at a density of 4,000 cells per well. After 24 h of incubation, the cells were cultured in DMEM + GlutaMAX medium, supplemented with 1% FBS (Sigma-Aldrich, 0001648078), and treated with 500 ng/mL of NBL1 (R&D, 955-DA) for 24 h. Following treatment, 50 μL of XTT mix from the Cell Proliferation Kit II (Roche, 11465015001, Germany) was added to the wells, and absorbance was measured at 480 and 690 nm after 3 h. The difference in absorbance between 480 and 690 nm was used to calculate the proliferation rate. The proliferation rate in NBL1 treated groups was calculated by dividing its absorbance by that of the untreated group.

### ELISA Assay

L6 myoblasts were seeded at a density of 3 × 10^5^ cells per well on FlexCell plates and subjected to either loading or unloading conditions for 24 h. The supernatant was then collected and centrifuged at 1000*g* for 5 min at 4 °C to remove cell debris. The supernatant was stored at −80 °C until all groups were ready for analysis. The concentration of NBL1 was measured using a rat NBL1 ELISA kit (BlueGene, E02N0097, China) according to the manufacturer’s instructions. Absorbance was recorded at 450 nm using a microplate reader, and the concentration of NBL1 was measured according to the concurrently generated standard curve.

### Statistics

Statistical analysis was performed using unpaired *t* tests or One-way ANOVA with Tukey’s multiple comparison post hoc test. Proteomics and ELISA samples were collected from three replicates. Migration and proliferation assays using human cells were conducted using cells from three different human donors (*n* = 3). Differences were considered statistically significant at a *P*-value of <0.05.

## Results

### Different Intensities of Static Loading on Myoblasts Induced Distinct Protein Expression Profiles

**Figure 1:**
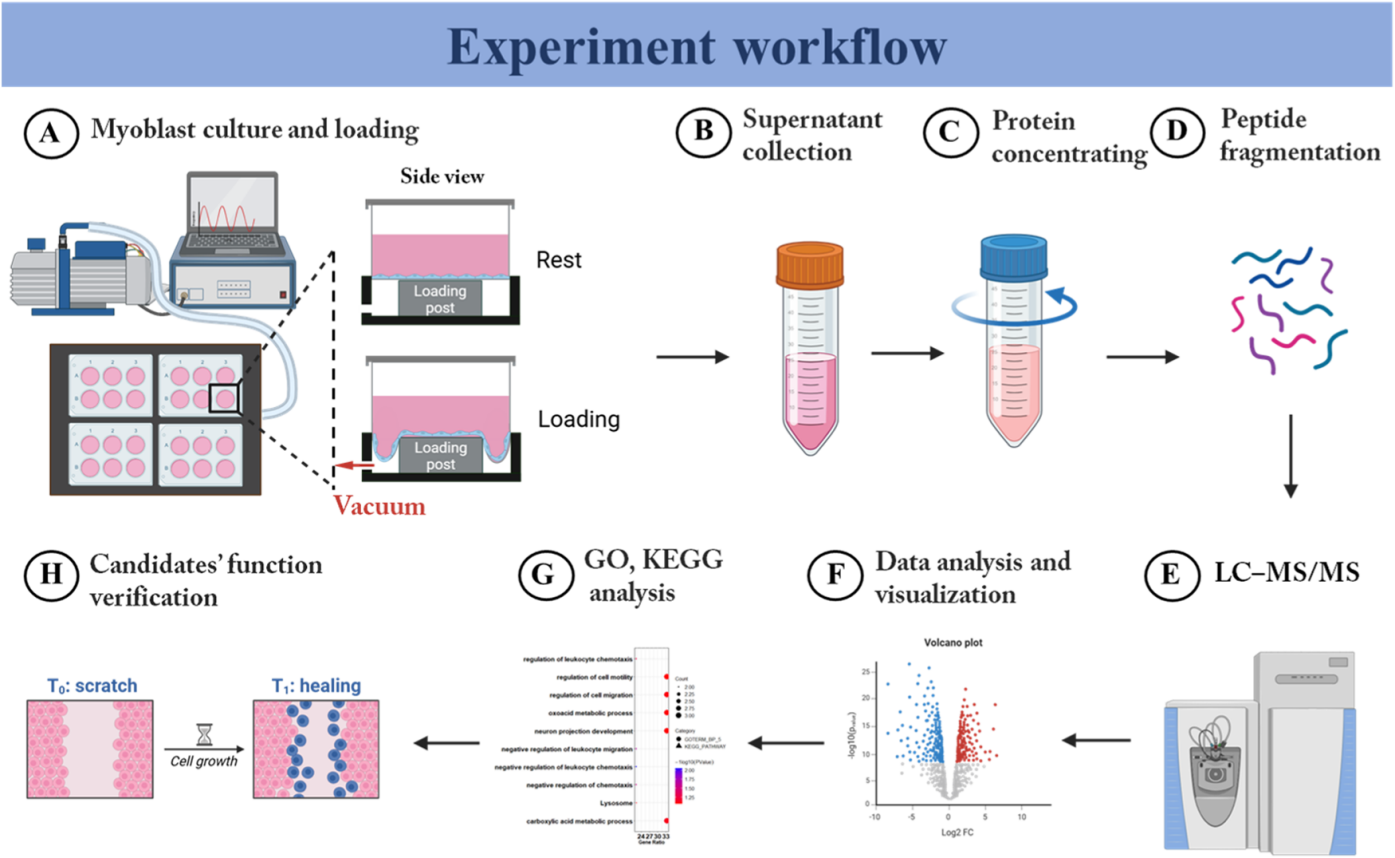
Experimental workflow of the study. (A) L6 myoblasts were loaded with different intensities for 24 h. (B) The supernatant was collected and (C) centrifuged to increase protein concentration. (D) Proteins were extracted and digested and (E) measured by LC-MS/MS. (F) Computational analyses identified the changes between the different loadings and control groups. (G) Gene ontology and KEGG pathways were analyzed. (H) Proteomics identified proteins’ function were verified in human tenocytes.

**Figure 2:**
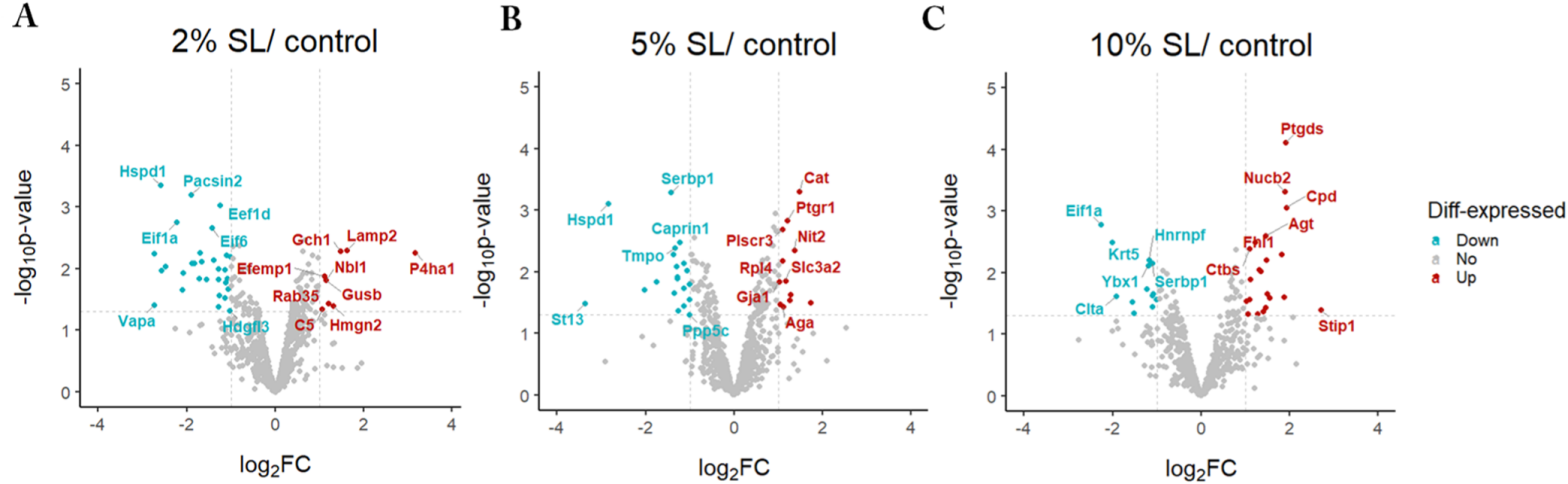
Different intensities of static loading on myoblasts induced distinct protein expression profiles. Volcano plots showing differentially secreted proteins among the 2, 5, and 10% loaded groups compared with the unloaded control group. The *x*-axis of (A), (B), and (C) illustrates the log_2_ fold change in protein abundance compared with the control group, while the *y*-axis represents the negative logarithm (base 10) of the *p*-value (*n* = 3, *p* < 0.05, log_2_ FC > 1 or log_2_ FC < −1). Upregulated proteins were labeled with red dots and downregulated proteins with blue dots. SL: static loading.

To identify the secretory proteins released by myoblasts in response to different intensities of static loading, proteomics was employed to quantify the types and abundance of secretions in the supernatant. See Figure 1 for the experimental workflow. In the 2, 5, and 10% statically loaded myoblasts, a total of 856, 815, and 759 proteins were identified, respectively. Significantly changed proteins between the loading and the unloaded control groups were defined based on fold changes (log_2_ Fold Change (FC) > 1 or log_2_ FC < −1) and statistical significance (*p*-value <0.05). This analysis revealed 9, 12, and 22 proteins with increased expression and 13, 20, and 30 proteins with decreased expression in the 2, 5, and 10% loading groups, respectively (Figure 2A,B,C).

### Differential Loading Intensities Induced Expression of Shared Proteins across Groups

**Figure 3:**
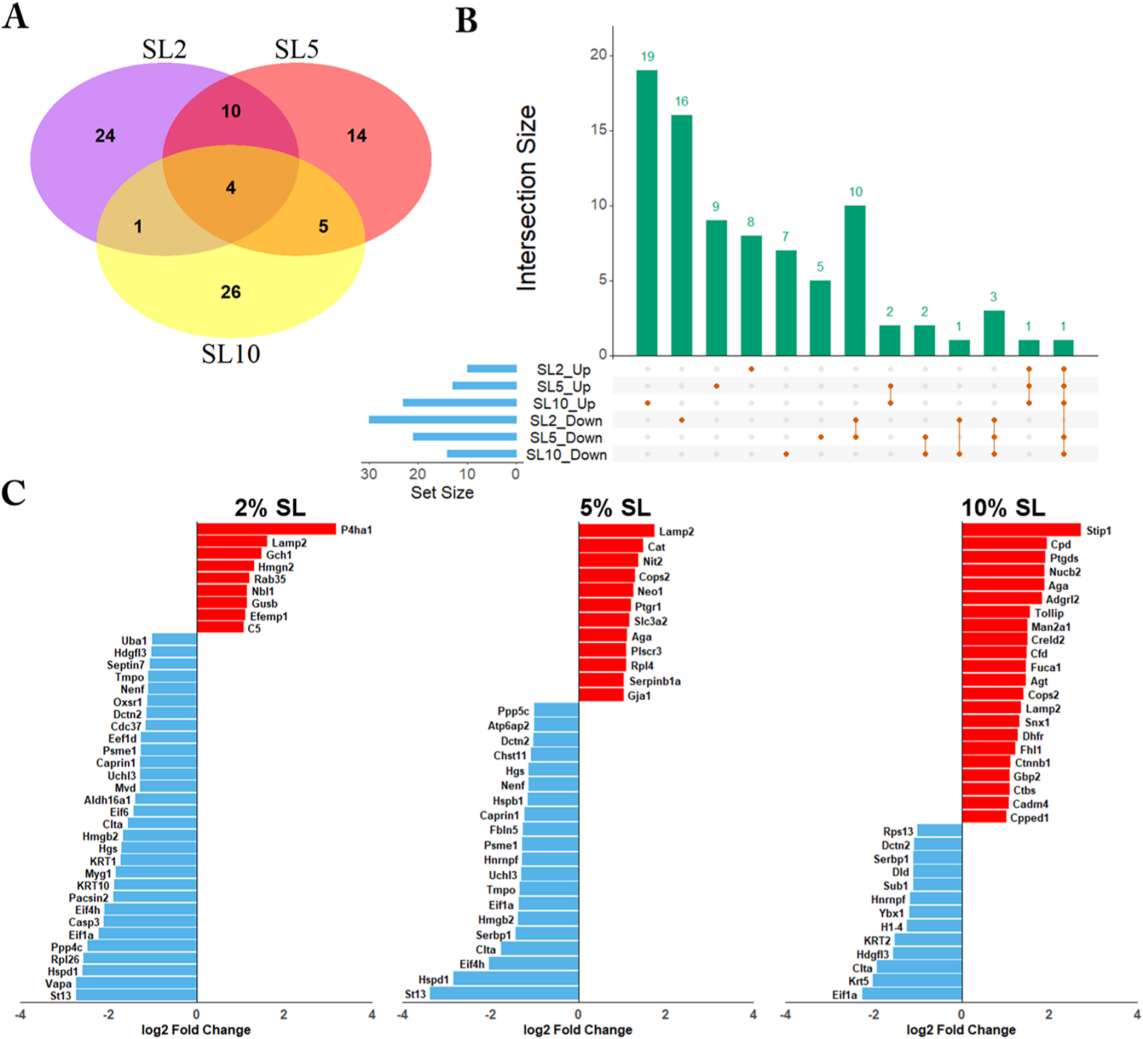
Differential loading intensities induced expression of shared proteins across groups. (A) Venn plot. (B) Upset plot illustrating the protein overlap among the 2, 5, and 10% static loading groups. The left transverse columns represent the total number of significantly expressed proteins in the labeled groups. A single orange dot, accompanied by the top column, indicates the number of proteins that are exclusively expressed within the labeled group. The orange line connecting two or more dots indicates the presence of overlapping proteins between these groups, while the top green columns indicate the counts of these overlapping proteins between these groups. (C) Display of significantly secreted proteins in 2, 5, and 10% static loading groups with their corresponding fold changes shown on *x*-axis. Upregulated proteins were labeled with red, and downregulated proteins were labeled with blue. SL: static loading; SL2/5/10:2%/5%/10% static loading; Up: upregulated proteins; Down: downregulated proteins.

Among the significantly altered proteins, there were some common proteins observed across different loading intensities (Figure 3A). In detail, Lamp2 was upregulated by 2, 5, and 10% loading, and Cops2 and Aga were upregulated in both the 5 and 10% loading groups. In the downregulated groups, the 2 and 5% loading groups shared 10 common downregulated proteins. Additionally, Hnrnpf and Serbp1 were common downregulated proteins in the 5 and 10% loading groups, and Hdgfl3 was common downregulated proteins in the 2 and 10% loading groups. Protein Clta, Dctn2, and Eif1a were consistently downregulated across all three loading intensities. Notably, there were no proteins that were upregulated at one intensity and downregulated at the other two intensities, and vice versa (Figure 3B). Figure 3C shows all of the significantly changed proteins across the loading and control groups. Interestingly, the number of upregulated proteins increased with the rise in intensity, while the number of downregulated proteins decreased.

### Potential of Differentially Expressed Proteins as Secretory Proteins

Our previous study showed that all three intensities of static loading did not induce cell death.^29^ Based on these findings, we investigated whether the significantly altered proteins originated from myoblast secretion. To this end, we employed SignalP-6.1 and SecretomeP-2.0 to predict the likelihood of these proteins being canonical or noncanonical secretory proteins. SignalP-6.1 identifies signal peptides indicative of canonical secretion, while SecretomeP-2.0 predicts noncanonical secretory proteins. The results indicated that 20 proteins possessed signal peptides predicted by SignalIP-6.1 or both SignalIP-6.1 and SecretomeP-2.0 (Table 1, column 1 labeled with A). Additionally, 12 proteins lacked signal peptides but were predicted to be noncanonical secretory proteins by SecretomeP-2.0 (Table 1, column 1 labeled with B). In total, 32 proteins were predicted to be secretory proteins.

**Table 1:** Potential of Differentially Expressed Proteins as Secretory Proteins^a^

			signalP-6.0	secretomeP	
	gene symbol	protein ID	signal peptide (Sec/SPI)	NN-score	groups
A	C5	P01031	0.9997	0.541	2% SL up
A	Efemp1	O35568	0.9997	0.756	2% SL up
A	Gch1	P22288	0.0012	0.526	2% SL up
A	Gusb	P06760	0.9991	0.646	2% SL up
A	Lamp2	P17046	0.9997	0.647	2, 5and 10% SL up
A	Nbl1	Q06880	0.9997	0.37	2% SL up
A	P4ha1	P54001	0.9997	0.808	2% SL up
A	Nenf	Q6IUR5	0.9996	0.93	2and 5% SL down
A	Aga	P30919	0.999	0.741	5and 10% SL up
A	Atp6ap2	Q6AXS4	0.9995	0.794	5% SL down
A	Fbln5	Q9WVH8	0.9997	0.814	5% SL down
A	Adgrl2	O88923	0.9996	0.292	10% SL up
A	Cadm4	Q1WIM1	0.9991	0.652	10% SL up
A	Cfd	P32038	0.9998	0.913	10% SL up
A	Cpd	Q9JHW1	0.9943	0.44	10% SL up
A	Creld2	Q4G063	0.9997	0.672	10% SL up
A	Ctbs	Q01460	0.997	0.695	10% SL up
A	Fuca1	P17164	0.9998	0.685	10% SL up
A	Nucb2	Q9JI85	0.9997	0.169	10% SL up
A	Ptgds	P22057	0.9998	0.894	10% SL up
B	Aldh16a1	Q3T1L0	0	0.683	2% SL down
B	Eif4h	Q5XI72	0	0.817	2and 5% SL down
B	Myg1	Q641W2	0	0.666	2% SL down
B	Pacsin2	Q9QY17	0	0.615	2% SL down
B	Clta	P08081	0	0.936	2, 5 and 10% SL down
B	Eef1d	Q68FR9	0	0.609	2% SL down
B	St13	P50503	0	0.759	2 and 5% SL down
B	Plscr3	Q6QBQ4	0	0.781	5% SL up
B	Chst11	P69478	0	0.81	5% SL down
B	Agt	P01015	0	0.818	10% SL up
B	Ybx1	P62961	0	0.73	10% SL down
B	Rps13	P62278	0	0.816	10% SL down

a Canonical and noncanonical secretory proteins analyzed by using Signal-6.0 and SecretomeP-2.0. A: Signal peptide predicted by Signal-6.0. B: Noncanonical secretory proteins predicted by SecretomeP-2.0. SL: static loading; Up: upregulated proteins; Down: downregulated proteins.

To explore the release mechanisms of the predicted nonsecretory proteins, we conducted an analysis using the Vesiclepedia database. Remarkably, all of these proteins were identified in the Vesiclepedia database as being secreted via extracellular vesicles in humans, rats, and/or mice (Table 2). These findings suggest that even proteins lacking classical secretion signals may be released via extracellular vesicles, providing insights into alternative secretion mechanisms under static loading conditions.

**Table 2:** Potential Presence of Proteins in Extracellular Vesicles^a^

gene symbol	protein ID	vesiclepedia	groups
Hmgn2	P18437	H	2% SL up
Rab35	Q5U316	H, R, M	2% SL up
Hspd1	P63039	H, R, M	2 and 5% SL down
Caprin1	Q5M9G3	H, M	2 and 5% SL down
Eif6	Q3KRD8	H, R, M	2% SL down
Hdgfl3	Q923W4	H	2 and 10% SL down
Hgs	Q9JJ50	H, R, M	2 and 5% SL down
Hmgb2	P52925	H, M	2 and 5% SL down
Rpl26	P12749	H, M	2% SL down
KRT1	P04264	H, M	2% SL down
KRT10	P13645	H, M	2% SL down
Mvd	Q62967	H	2% SL down
Ppp4c	Q5BJ92	H, M	2% SL down
Psme1	Q63797	H, M	2 and 5% SL down
Oxsr1	A0A8I5ZNK2	H, R, M	2% SL down
Septin7	Q9WVC0	H, R, M	2% SL down
Casp3	P55213	H, M	2% SL down
Cdc37	Q63692	H, M	2% SL down
Dctn2	Q6AYH5	H, M	2, 5and 10% SL down
Eif1a	Q6VV72	H, M	2, 5and 10% SL down
Tmpo	Q62733	H	2 and 5% SL down
Uba1	Q5U300	H, R	2% SL down
Uchl3	Q91Y78	H, R, M	2 and 5% SL down
Vapa	Q9Z270	H, M	2% SL down
Neo1	P97603	H, M	5% SL up
Cat	P04762	H, R	5% SL up
Cops2	P61203	H, R, M	5 and 10% SL up
Nit2	Q497B0	H, R, M	5% SL up
Gja1	P08050	H, M	5% SL up
Ptgr1	P97584	H, M	5% SL up
Serpinb1a	Q4G075	R, M	5% SL up
Slc3a2	Q794F9	H, R, M	5% SL up
Rpl4	P50878	H, M	5% SL up
Hnrnpf	Q794E4	H, M, R	5 and 10% SL down
Ppp5c	P53042	H, M	5% SL down
Serbp1	Q6AXS5	H, R, M	5 and 10% SL down
Fhl1	Q9WUH4	H, M	10% SL up
Cpped1	Q66H71	H, M	10% SL up
Dhfr	Q920D2	H, R, M	10% SL up
Gbp2	Q63663	H, R, M	10% SL up
Man2a1	P28494	H, M	10% SL up
Snx1	Q99N27	H, M	10% SL up
Stip1	O35814	H, R, M	10% SL up
Tollip	A2RUW1	H, R, M	10% SL up
Krt5	Q6P6Q2	H, R, M	10% SL down
Dld	Q6P6R2	H, R, M	10% SL down
H1–4	P15865	H, R, M	10% SL down
KRT2	P35908	H, M	10% SL down
Sub1	Q63396	H, R, M	10% SL down

a The potential presence of these proteins in extracellular vesicles by verifying their existence in the database of Vesiclepedia. H: *Homo sapiens*; R: *Rattus norvegicus*; M: *Mus musculus*. SL: static loading; Up: upregulated proteins; Down: downregulated proteins.

### Enriched Biologic Processes and Pathways of Differentially Expressed Proteins in Response to Static Loading of Myoblasts

**Figure 4:**
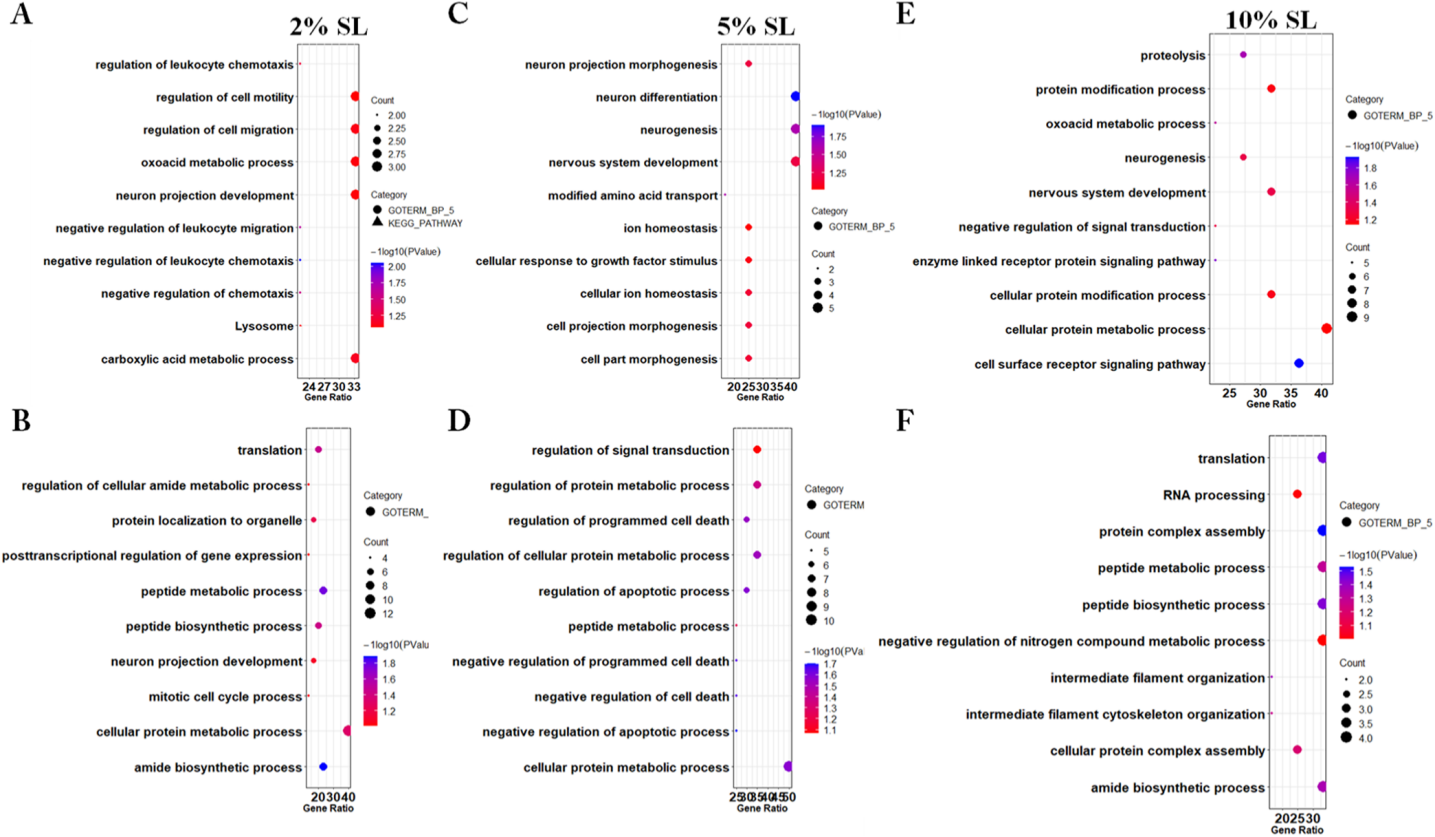
Enriched biological processes and pathways of differentially expressed proteins in response to the static loading of myoblasts. Bubble charts displayed the top 10 most significantly altered biological processes in GO and pathways in the KEGG for up- and downregulated proteins within the 2% (A, B), 5% (C, D), and 10% (E, F) static loading groups, respectively. The *x*-axis represents the ratio of the involved genes in each process compared to the total genes in the given list, while the *y*-axis represents the KEGG/GO terms. The size of the dots corresponds to the number of genes, and the color indicates the *p*-value. The color from blue to red represents a smaller *p*-value to a larger one. SL: static loading.

We utilized the DAVID database to analyze enriched biological processes and pathways for the significantly altered proteins. The top 10 enriched Biological Processes (BP) in Gene Ontology and KEGG pathways, based on the gene ratio (the ratio of the number of genes associated with a specific GO term or KEGG pathway relative to the total number of input genes), are shown in Figure 4A–F.

In myoblasts subjected to 2% static loading, the upregulated proteins were enriched for functions related to cell migration and motility, including proteins such as NBL1, C5, and EFEMP1. Additionally, processes related to the oxoacid metabolic process, neuron projection development, carboxylic acid metabolic process, and leukocyte chemotaxis were identified (Figure 4A). In the list of downregulated proteins, the top enriched processes were related to cellular protein metabolic processes, based on the gene ratio. Most other terms were also related to peptide and protein biosynthesis and metabolism. Notably, some downregulated proteins were involved in neuron projection development (Figure 4B). In summary, 2% static loading stimulates the secretion of proteins that are associated with increased cell migration, while downregulated proteins were associated with protein biosynthetic-related processes.

In myoblasts subjected to 5% static loading, the up-regulated proteins were enriched for processes related to neurons. Additionally, enriched processes included ion homeostasis and cell part morphogenesis (Figure 4C). In the list of downregulated proteins, the most enriched processes were associated with protein metabolism, signal transduction, and regulation of cell death (Figure 4D). In summary, 5% static loading stimulates the secretion of proteins that may play an important role in the nervous system, while downregulated proteins are crucial for regulating protein metabolism.

In myoblasts subjected to 10% static loading, the upregulated proteins were enriched for processes related to cell surface receptor signaling pathways, protein metabolism and

modification, and the nervous system (Figure 4E). In the list of downregulated proteins, most processes were related to RNA and protein synthesis, with some processes associated with

intermediate filament organization (Figure 4F). In summary, 10% static loading stimulates the secretion of some proteins associated with protein metabolism and modification processes

while simultaneously downregulating some other protein synthesis-related proteins.

### 2% Static Loading of Myoblasts Increased NBL1 Secretion, Promoting Tenocyte Migration

**Figure 5:**
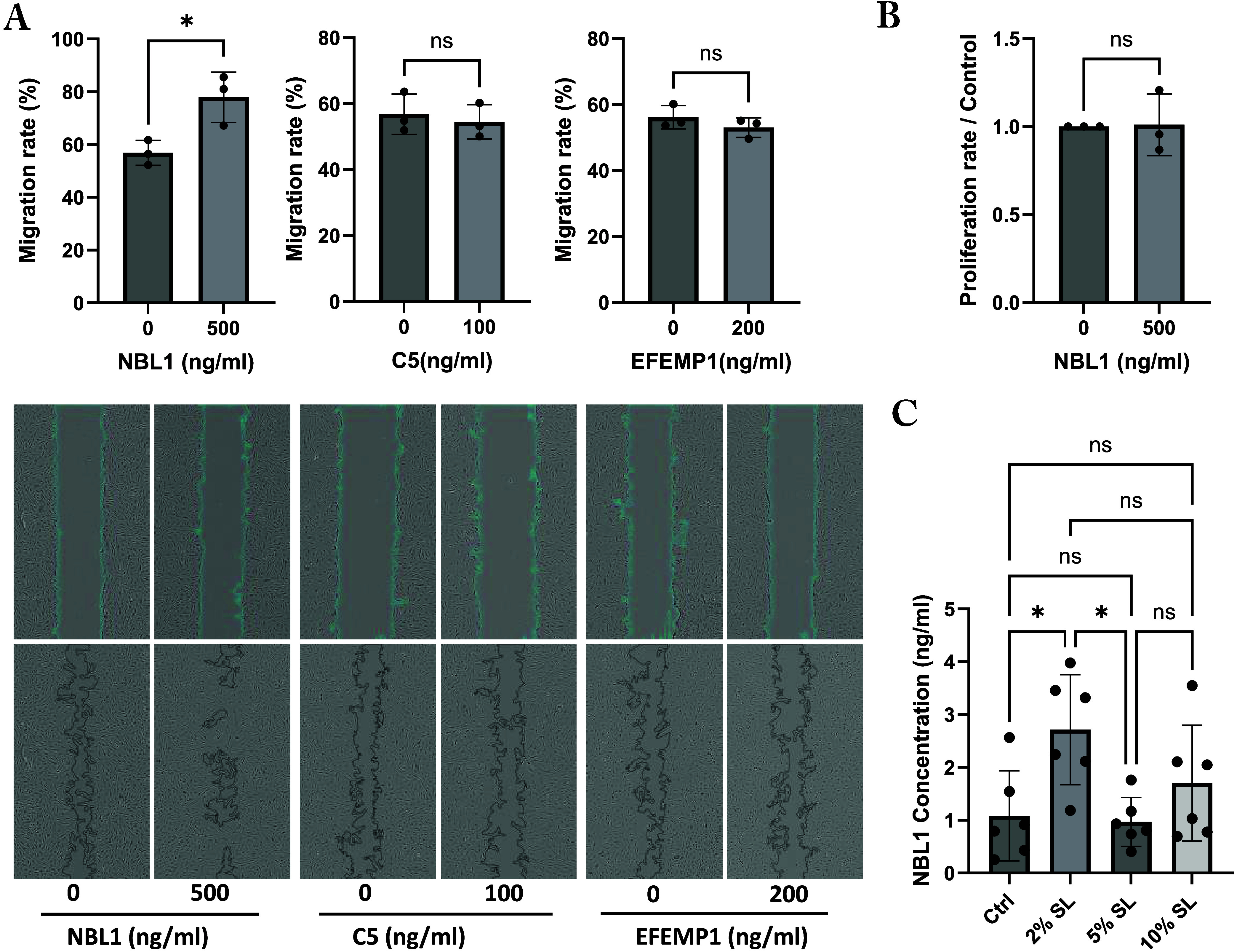
2% static loading of myoblasts increased NBL1 secretion, promoting tenocyte migration. (A) The effects of NBL1, C5, and EFEMP1 on tenocyte migration. Migration rate of 100% indicates complete closure of wound. (B) The effect of NBL1 on tenocyte proliferation. (C) NBL1 concentration in the supernatant in response to varying static loading intensities using ELISA. Ctrl: myoblast without loading. 2, 5, and 10% SL represent static loading of myoblast at different intensities. Results shown are mean ± SD (*n* = 3 (human donors), *n* = 6 (myoblast supernatant)).  Ns: not significantly different, *P < 0.05. SL: static loading.

Gene ontology analysis identified NBL1, C5, and EFEMP1 as proteins related to cell migration and motility. To investigate whether these proteins affect tenocyte migration, we treated human tenocytes with human recombinant NBL1, C5, and EFEMP1. Our findings showed that NBL1, at a concentration of 500 ng/mL as used in previous studies,^32,33^ significantly increased tenocyte migration, while C5 (100 ng/mL^34,35^) and EFEMP1 (200 ng/mL^36^) had no effect (Figure 5A). Importantly, NBL1 did not influence tenocyte proliferation, indicating that the increase in migration was independent of the proliferation (Figure 5B). Additionally, by measuring NBL1 levels in the supernatant, we confirmed that 2% static loading of myoblasts results in increased secretion as compared with control, 5 and 10% static loading (Figure 5C).

## Discussion

Our proteomic data demonstrated that different intensities of static loading on myoblasts lead to the secretion of distinct profiles of myokines. Among these myokines, our primary aim was to identify proteins with wound-healing properties relevant to tenocytes. Our results showed that 2% static loading of myoblasts significantly increased the secretion of NBL1, C5, and EFEMP1. Gene ontology analysis implicated these proteins in regulating cell migration, which is a crucial process in wound healing. By adding exogenous recombinant NBL1, C5, and EFEMP1 to tenocytes, we discovered that NBL1 enhances tenocyte migration but not proliferation, whereas C5 and EFEMP1 do not exhibit this function.

We identified 856, 815, and 759 supernatant proteins in myoblasts subjected to 2, 5, and 10% static loading, respectively. This range aligns with previous studies reporting between 236 and 1073 proteins in skeletal muscle cell supernatants.^37−41^ The varying number of identified supernatant proteins is likely due to differences in proteomic workflows, cell sources, applied mechanical loads, cell differentiation stages, culture media, and other factors. Nevertheless, the number of proteins we identified is within the reported range. The observed decrease in the total number of secreted proteins with increasing static loading intensity may suggest an intensity-dependent effect. Our previous study supports this, showing that 2% static loading enhances RNA synthesis in myoblasts, while 10% static loading decreases it.^29^

Of the 81 differentially released proteins, 49 were predicted to be nonsecretory and were analyzed using the extracellular vesicle (EV) database. Remarkably, all were identified as likely secreted via EVs, consistent with studies showing muscle cells release proteins through microvesicle shedding.^38,42^ For instance, skeletal muscle-derived EVs have been shown to transport glycolytic enzymes to facilitate muscle-to-bone crosstalk.^43^ Similarly, a study reported that the injection of skeletal muscle-derived EVs from exercised mice exerts protective effects similar to those of exercise itself.^44^ These findings suggest that skeletal muscle communicates with other tissues via EVs. This presents an intriguing pathway for investigating intercellular communication, in particular, the role of EVs. When the function of specific proteins secreted via EVs using exogenous recombinant proteins is examined, it is crucial to determine whether these recombinant proteins can be internalized by cultured cells or tissues in the same manner as their natural incorporation within EVs. However, since our primary interest was in migration-regulating proteins, GO analysis identified NBL1, C5, and EFEMP1 as relevant proteins that have signal peptides. Therefore, the focus of this study was not on the further investigation of EVs.

We found that static loading of myoblasts upregulated the secretion of proteins that have potential functions in the regulation of leukocyte chemotaxis and migration and cytokine production, indicating their control over the movement of immune cells. Additionally, the secreted protein’s negative regulation of apoptotic processes, cell death, and programmed cell death highlights mechanisms that prevent unnecessary cell loss. These upregulated proteins are also integral in modulating enzyme-linked receptor signaling pathways, which are crucial for cellular communication and responses to external stimuli. Notably, proteins upregulated by 2 and 10% static loading have the potential to positively regulate the nervous system. This finding aligns with prior studies indicating that physical exercise induces the release of molecules involved in neuronal survival, differentiation, plasticity, and neurogenesis.^45,46^ Therefore, these proteins hold promise for researchers in related fields exploring their therapeutic potential.

Among the differentially expressed proteins, our focus was on secreted proteins relevant to wound healing, specifically those with the potential to induce tenocyte migration. In our previous study, we identified IGF-1 as a secreted factor from 2% static loading of myoblasts that was responsible for inducing tenocyte proliferation.^26^ However, RNA-sequencing of loaded myoblasts did not reveal any factors responsible for inducing tenocyte migration under the same conditions.^26^ Interestingly, proteomic analysis of the secretome from 2% of statically loaded myoblasts identified NBL1, C5, and EFEMP1 as proteins involved in cell migration. NBL1, also known as DAN, acts as an antagonist of bone morphogenetic proteins (BMPs), more specifically BMP-2, -4, -7,^47,48^ by binding to them and inhibiting their interaction with receptors, potentially influencing growth and development processes. One study has shown that NBL1 is prominently expressed in corneal stromal cells during wound healing and reduces corneal fibrosis and scar formation in mice and cultured human corneas.^32^ Given the similarities between tenocytes and corneal stromal cells as connective tissue cells, it could be speculated that NBL1 might also facilitate tendon healing.

Cell migration is one of the earliest responses to tissue injury and plays a crucial role in wound healing. While NBL1 has been shown to inhibit migration in some contexts,^49^ its role in tenocyte migration has not been previously investigated. BMP-2, -4, and -7, which are antagonized by NBL1, are known to induce both migration and proliferation, including in fibroblasts.^50−52^ Our results show that the exogenous administration of NBL1 increases tenocyte migration without affecting proliferation. Interestingly, our findings suggest that NBL1 promotes tenocyte migration independently of exogenous BMP administration. Although circulating BMP-2 and -4 have been detected in serum,^51^ their concentration in our experimental setup using only 1% serum is likely insignificant. Previous studies have shown that NBL1 also can interact with specific cell surface receptors, including those involved in integrin signaling and growth factor receptor pathways.^49^ These interactions may trigger downstream signaling cascades that regulate cytoskeletal dynamics, focal adhesion turnover, and the secretion of matrix metalloproteinases (MMPs), all of which are essential for cell migration. However, the intriguing downstream signaling pathway of NBL1 was not the focus of this study and has, therefore, not been extensively studied.

Research into the migration-regulatory role of EFEMP1 has predominantly focused on cancer^53−57^ and other pathological conditions,^58,59^ indicating that EFEMP1’s effect on migration is context-dependent and influenced by the specific pathological microenvironment. Similarly, C5, is primarily recognized for its role in directing immune cell migration toward sites of inflammation,^60,61^ and has been associated with migration in certain pathological contexts, such as cancers.^62,63^ Given the distinct physiological environment of tenocytes compared to that of immune or cancerous cells, it is reasonable that EFEMP1 and C5 did not exert a significant influence on tenocyte migration in this study.

Based on these findings that NBL1 increased tenocyte migration, whereas C5 and EFEMP1 did not, it can be speculated that the tenocyte migration observed in response to secreted factors from 2% statically loaded myoblasts as previously demonstrated,^26^ is at least partly attributed to NBL1 secretion. However, the exact role of NBL1 on tenocytes under the influence of other secreted proteins following 2% static loading of myoblasts has not been investigated. Our previous and current results suggest that static loading of myoblasts, particularly at 2% mechanical loading, results in favorable outcomes for tendon healing by increasing both proliferation and migration of tenocytes via IGF-1 and NBL1, respectively. Nevertheless, further studies are essential to determine whether this type and intensity of loading can be effectively translated into an exercise regimen for treating tendon injuries.

While this study provides evidence for the role of NBL1 in tenocyte migration, the mechanistic effect of NBL1 remains unclear. Future research should focus on elucidating the specific receptors and unraveling downstream signaling pathways activated by NBL1 in tenocytes. One limitation is that our experiments were performed using 2D models; however, to further explore secreted proteins from myoblasts in accelerating tendon wound healing or enhancing healing outcomes, our future work should utilize a 3D tendon formation model. Another limitation is that we used myoblast instead of myotubes; the primary reason for using single-cultured L6 myoblasts rather than myotubes was the challenge of inducing homogeneous myotube formation, which could result in variability in the experimental outcomes. Myoblasts offer a more consistent and reproducible secretory profile, as their secretome is not influenced by high variability in stages of differentiation or fusion, ensuring greater reliability in our analyses. However, in the experimental setup used, including serum starvation, we could see that the L6 cells began a myotube transition, indicated by reduced MyoD1 expression and increased MYHC1 and MyoG.^29^ Although the secretome may differ between myoblasts and myotubes during mechanical loading, our findings using myoblasts closely align with in vivo exercise models. Specifically, our experimental setup demonstrated that higher intensities of loading activate AMPK and inhibit mTOR signaling, trends that mirror those observed in muscle tissue during in vivo exercise.^29^ This consistency validates our choice of myoblasts for the mechanical loading experiments and supports their relevance as a model.

## Conclusions

In conclusion, our study identified NBL1, C5, and EFEMP1 as myokines induced by 2% static loading, which are implicated in regulating cell migration. Notably, we demonstrated for the first time that NBL1 can enhance tenocyte migration. Additionally, our investigation revealed several other myokines involved in protein metabolic processes, the mitotic cell cycle, the regulation of leukocyte chemotaxis, and nervous system development. These findings provide a basis for investigating the paracrine or endocrine roles of these factors in muscles and other tissues. Moreover, given the potential release of some proteins in our myoblast supernatant via EVs, investigating these EVs separately offers an alternative approach to explore their effects on tendon wound healing.

## Data Availability

The mass spectrometry proteomics data have been deposited to the PRIDE Archive (http://www.ebi.ac.uk/pride/archive/) via the PRIDE partner repository with the data set identifier PXD055477.
